# Heritability of Caries Scores, Trajectories, and Disease Subtypes

**DOI:** 10.1177/0022034519897910

**Published:** 2020-01-06

**Authors:** S. Haworth, A. Esberg, P. Lif Holgerson, R. Kuja-Halkola, N.J. Timpson, P.K.E. Magnusson, P.W. Franks, I. Johansson

**Affiliations:** 1 Bristol Dental School, University of Bristol, Bristol, UK; 2 Medical Research Council Integrative Epidemiology Unit, Department of Population Health Sciences, Bristol Medical School, University of Bristol, Bristol, UK; 3 Section of Cariology, Department of Odontology, Umeå University, Umeå, Sweden; 4 Section of Pedodontics, Department of Odontology, Umeå University, Umeå, Sweden; 5 Department of Medical Epidemiology and Biostatistics, Karolinska Institutet, Stockholm, Sweden; 6 Department of Clinical Sciences, Genetic and Molecular Epidemiology Unit, Lund University, Malmö, Sweden; 7 Department of Nutrition, Harvard T. H. Chan School of Public Health, Boston, MA, USA

**Keywords:** twins, longitudinal, genetics, cluster analysis, dental caries susceptibility, epidemiology

## Abstract

Previous studies report that dental caries is partially heritable, but there is uncertainty in the magnitude of genetic effects and little understanding of how genetic factors might influence caries progression or caries subtypes. This study aimed to estimate the relative importance of genetic and environmental factors in the etiology of different caries outcomes using a twin-based design. Analysis included up to 41,678 twins in the Swedish Twin Register aged 7 to 97 y, and dental data were obtained from preexisting dental records. The outcome measures were 1) summary indices of caries experience, 2) parameters representing trajectory in caries progression derived from longitudinal modeling, and 3) caries scores in groups of biologically similar tooth surfaces derived from hierarchical clustering of tooth surfaces (termed *caries clusters*). Additive genetic factors explained between 49.1% and 62.7% of variation in caries scores and between 50.0% and 60.5% of variation in caries trajectories. Seven caries clusters were identified, which had estimates of heritability lying between 41.9% and 54.3%. Shared environmental factors were important for only some of these clusters and explained 16% of variation in fissure caries in molar teeth but little variation in other clusters of caries presentation. The genetic factors influencing these clusters were only partially overlapping, suggesting that different biological processes are important in different groups of tooth surfaces and that innate liability to some patterns of caries presentation may partially explain why groups of tooth surfaces form clusters within the mouth. These results provide 1) improved quantification of genetic factors in the etiology of caries and 2) new data about the role of genetics in terms of longitudinal changes in caries status and specific patterns of disease presentation, and they may help lay the foundations for personalized interventions in the future.

## Introduction

Dental caries is one of the most prevalent chronic diseases worldwide, with approximately 2,500 million adults and 500 million children affected by untreated caries (Vos et al. 2016; Kassebaum et al. 2017). It is a complex disease where the clinical presentation is thought to result from an interplay between genetic and environmental components, and the theory of a genetic contribution to caries susceptibility was proposed by animal studies >70 y ago (Hunt et al. 1944).

Since then, a number of studies have investigated the relative importance of genetic and environmental factors in the etiology of caries with families and twins reared together or apart (Boraas et al. 1988; Conry et al. 1993; Bretz, Corby, Hart, et al. 2005; Bretz, Corby, Schork, et al. 2005; Bretz et al. 2006; Wang et al. 2010; Shaffer, Feingold, et al. 2012; Shaffer, Wang, et al. 2012; Shaffer et al. 2015), reporting that genetic factors explain between 20% and 65% of variation in disease scores. Studies also report a genetic influence on other outcomes that are potentially associated with total disease level, such as tooth morphology, presence of untreated disease, number of affected occlusal surfaces, oral bacteria profiles or acquisition, and sweet taste preference (Kabban et al. 2001; Race et al. 2006; Keskitalo et al. 2007; Rintakoski et al. 2010; Gomez et al. 2017). The wide range of heritability estimates may relate to chance (given the relatively small sample sizes included in several studies) or to differences among cohorts with respect to causal or caries-modifying factors as well as access to dental care. Recent studies suggest that, even within a single cohort, the heritability of caries might vary with tooth type, tooth arch segment, part of the tooth, or sex (Wang et al. 2010; Shaffer, Wang, et al. 2012; Shaffer et al. 2015). One example of this is the unconditional clustering of tooth surfaces by caries status into biological units that are reported to have differential genetic susceptibility (Shaffer et al. 2013). Finally, a recent study challenges the interpretation of a moderate genetic basis for caries susceptibility and argues that genetic factors have low relevance as compared with environmental factors in preschool children (Silva et al. 2019). Thus, there is a need for additional heritability estimates in a large-scale, population-representative sample, which would help clarify the relative importance of genetic and environmental influences for different patterns of caries presentation. Knowledge of which caries traits are most heritable and biologically informative might also help guide the design and interpretation of genetic association studies investigating the role of specific allelic variants in the etiology of caries (Nibali et al. 2017; Shungin et al. 2019), where the variants identified to date explain <2% of variation in caries scores (Appendix).

One way to approach these questions is to perform large-scale analysis with the twin study design (Boomsma et al. 2002) and high-quality register data. The Swedish Twin Register (Magnusson et al. 2013), which was initiated in the early 1960s, is the largest population-based register of twin pairs with known zygosity. It contains >85,000 twin pairs, and annually all incident twins are recruited in early school age. Of the twin pairs, approximately 25% are monozygotic, with the remainder being same- or opposite-sex dizygotic.

This study set out to explore the relative importance of genes and environment change with age, to test how genetic factors influence trajectory in caries scores, and to test whether caries clusters represent etiologically distinct diseases based on the heritable contributions to each cluster.

## Methods

The project is reported in accordance to the STROBE guidelines (Strengthening the Reporting of Observational Studies in Epidemiology) for cohort studies and received ethical approval (Appendix).

### Participants and Data Collection

The study included twins in the Swedish Twin Register (https://ki.se/en/research/the-swedish-twin-registry) for whom information on caries status could be retrieved from Public Health Care records by linking the 12-digit unique person identification numbers.

### Zygosity Determination

Information on zygosity was determined by a validated test based on 46 single-nucleotide polymorphism markers where available (Magnusson et al. 2013), by an intrapair similarity algorithm, or by being opposite sex. Approximately 12% of all complete pairs had their zygosity determined by DNA-based tests. Twin pairs with unknown zygosity were excluded from the analysis.

### Derivation of Caries Scores

Dental examination in the Swedish Public Health Care includes visual inspection with good light, a mirror, tactile examination with a dental probe, and bitewing radiographs and electronic recording of the status of each tooth surface (mesial, distal, buccal, lingual, and occlusal). Summary indices representing the number of decayed, missing, and filled surfaces (DMFS) and decayed, missing, and filled proximal surfaces (DMFS_proximal_), including third molar teeth, were retrieved. Teeth missing due to caries were imputed as 4 surfaces for anterior teeth and 5 surfaces for posterior teeth. Teeth missing for other reasons were not included in scores. To explore possible biasing effects from tooth loss, scores were also created with decayed and filled surfaces only (DFS and DFS_proximal_). To help minimize disagreement among practitioners, initial caries signs were not included in the summary scores.

### Derivation of Caries Trajectory Scores

Caries status was obtained from each visit where the participant had a full mouth examination. Longitudinal analysis of incident caries was restricted to twins where both twins in a pair had ≥3 examinations with at least 2 y of dental follow-up. A linear mixed model approach was used to model changes in caries scores over time, accounting for multiple measures. Raw caries scores were regressed on age at examination, with fixed effects for sex and age at examination and with random intercepts and slopes for each twin. The random effects for slope (how quickly caries scores change in a twin relative to the overall linear change with age) were estimated for each participant via the “mixed” function of Stata 15. This analysis assumes that changes in caries scores are linear during the duration of follow-up. To relax this assumption, additional analysis was performed via a nonparametric growth curve method based on the SITAR approach (superimposition by translation and rotation; Cole et al. 2010). This models population-average nonlinear changes in a trait over time with spline regression and then compares how any individual’s trajectory deviates from the population average, by fitting up to 3 random effects. For this analysis, a random effect representing velocity (i.e., the extent by which changes in caries scores occur more or less rapidly than the population average) was estimated for each participant via the SITAR package (version 1.1.1, March 2019) implemented in R (version 3.5.3, March 2019).

### Clustering and Derivation of Per-Cluster Scores

For most participants, tooth status data at the surface level were available. After exclusion of third molars, each tooth surface was classified as sound (coded as 0) or caries affected (signs of manifest caries, restoration, or missing tooth; coded as 1). Hierarchical clustering with the Ward method and squared Euclidean distance measures was used to identify groups among the 128 tooth surfaces, called *clusters* in the Results section. Cluster definitions were generated in a data set including a randomly selected twin from each twin pair and validated in the remaining twins. After validation, per-cluster scores were derived for use as quantitative traits in subsequent analysis, representing the sum of the surface-level codes for all tooth surfaces in that cluster.

### Statistical Modeling

For each caries trait, quantitative genetic models (Kohler et al. 2011) were fitted with data from monozygotic and dizygotic twin pairs and same- and opposite-sex twin pairs. These models assume that caries scores are partially correlated in twin pairs, and this correlation is partially due to genetic factors and partially due to shared environmental factors that affect both twins in a pair. To distinguish between these 2 sources of correlation, the models examine monozygotic twin pairs (who share ~100% of their nuclear DNA) and dizygotic twin pairs (who on average share ~50% of their nuclear DNA). This distinction allows the models to estimate the variation in caries scores that are attributable to additive genetic effects (termed *variance component A*) and shared environmental factors (termed *variance component C*). Finally, although caries scores are partially correlated in twin pairs, they are not perfectly correlated even in monozygotic twin pairs, which implies the existence of unique environmental factors that affect only 1 twin in a pair. It is therefore possible to estimate how much variation in caries scores is due to nonshared environmental factors, which is combined with the remaining unexplained variance (error) in the model (termed *variance component E*). These models (termed *ACE models*) were fitted with the software tool OpenMX (Neale et al. 2016; version 2.13.2) implemented in R (version 3.5.3, March 2019).

The primary analysis incorporated adjustment for age, age squared, and birth year for all traits. To assess changes in the heritability of DMFS with age, additional analysis was performed that treated age as a modifier variable (see Appendix for a detailed description of the statistical modeling).

## Results

### Final Sample Included in Analysis

Dental data were available for twins living in 13 of 20 counties in Sweden and for dental examinations between 1999 and 2015. Caries data could be retrieved for 52,062 people in 26,784 pairs, of which 1,506 participants lacked a pair mate (2.9%) and 4,439 twin pairs (16.6%) were of unknown zygosity. The final study group therefore included 20,839 complete twin pairs with known zygosity and caries data (Table). The number of adult twins (≥20 y) was similar to the number of children and teenagers. Longitudinal analysis included 26,414 twins and a total of 138,566 separate dental examinations. On average, twins had a full mouth examination every 1.2 y, had 5.2 visits during study follow-up (range, 3 to 16), and had 6.3 y of longitudinal follow-up (range, 2.0 to 13.1 y). A summary of caries status in monozygotic and dizygotic twins is show in Appendix Tables 1A and 1B. All caries indices and trajectories were more closely correlated in monozygotic twin pairs than in dizygotic twin pairs, justifying the decision to fit quantitative genetic models (Appendix Table 2).

**Table.** Demographic Information for the Final Sample Included in Each Analysis.

			Mean (range)				
Analysis	Twin Pairs (MZ)	Outcomes,^a^ *n*	DMFS	DMFSa	DFS	DFSa	Age
Cross-sectional analysis							
All ages^b^	20,839 (6,370)	41,678	17.5 (0 to 148)	7.1 (0 to 64)	9.3 (0 to 129)	3.8 (0 to 56)	25.3 (7 to 97)
≥20 y	9,830 (3,414)	19,660	34.7 (0 to 148)	14.5 (0 to 64)	18.1 (0 to 129)	7.8 (0 to 56)	38.7 (19 to 97)
<20 y	11,192 (2,996)	22,384	2.2 (0 to 75)	0.6 (0 to 41)	1.4 (0 to 74)	0.3 (0 to 39)	13.4 (7 to 20)
Longitudinal analysis^c^	13,207 (4,153)	138,566	15.7 (0 to 148)	6.4 (0 to 64)	8.5 (0 to 129)	3.4 (0 to 56)	24.5 (9 to 97)
Cluster analysis (age ≥20 y)	9,481 (3,262)	113,772	—	—	—	—	40.1 (19 to 97)

DMFSa, DMFS_proximal_; DFSa, DFS_proximal_; MZ, monozygotic.

a Number of observed outcome measures included in each statistical model.

b The sample sizes by age group do not add to the total because twin pairs were excluded when they fell into different age groups with an age difference >1 y at their dental visit.

c For longitudinal analysis, all measures are given for the most recent dental examination.

### Heritability of DMFS Scores in Cross-sectional Analysis

**Figure 1.**
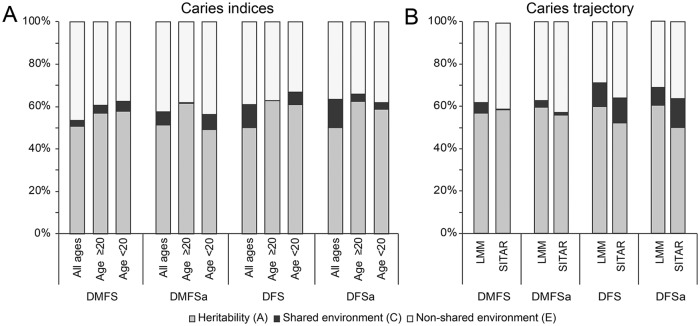
Results of variance decomposition for caries indices and caries trajectories. Each bar represents a caries trait, and the stacked components represent the relative contributions of components A (additive genetic factors), C (shared environmental factors), and E (nonshared environmental factors) to variation in that trait. DMFSa and DFSa refer to DMFS_proximal_ and DFS_proximal_, respectively. Results of (**A**) cross-sectional analysis and (**B**) trajectory modelling. See Appendix Tables 3 and 4 for confidence intervals and *P* values. DFS, decayed and filled surfaces; DMFS, decayed, missing, and filled surfaces.

In cross-sectional analysis, the estimates of heritability ranged between 49.1% and 62.7%, with similar estimates for DMFS and DMFS_proximal_ and for DFS and DFS_proximal_ (Fig. 1A, Appendix Table 3). In general, heritability estimates had a similar interpretation in adults and young people, with slightly higher point estimates in adults. Shared environmental factors explained <15% of variation in caries scores in all cross-sectional analyses, although there was variation among caries indices and age groups. When compared with shared environmental factors, nonshared environmental factors were consistently important for all traits and in different age groups, explaining between 33.1% and 46.4% of variation in caries indices.

### Heritability of Caries Trajectory Parameters

In longitudinal analysis, the estimates of heritability for caries trajectory parameters ranged between 50.0% and 60.5%. Results from all 4 caries indices had a similar interpretation, and results were comparable from the linear mixed model and SITAR approaches (Fig. 1B, Appendix Table 4). Shared environmental factors explained relatively little variation in trajectory parameters, while nonshared environmental factors were important for all 4 caries indices.

### Age-Moderated Analysis

**Figure 2.**
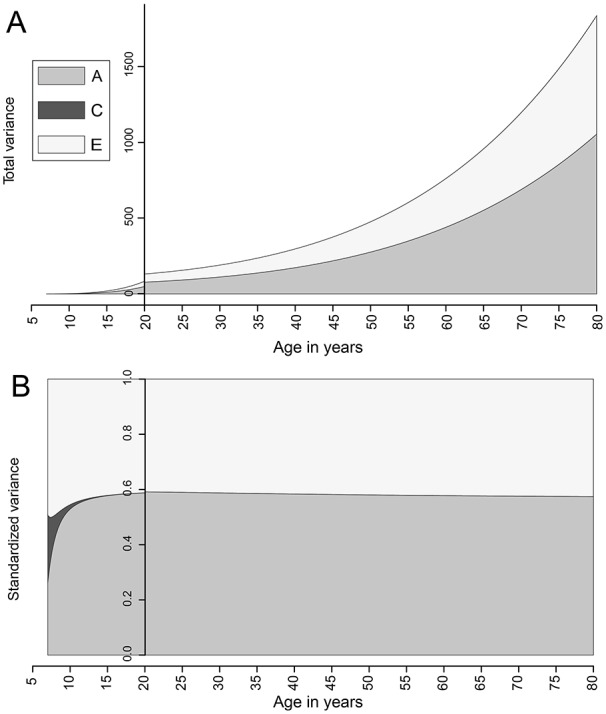
Change in the absolute and relative importance of variance components with age for DMFS. The plots show fitted values of the total variance (upper panel) and standardized variance (lower panel) attributable to components A (additive genetic factors), C (shared environmental factors), and E (nonshared environmental factors) in age-moderated cross-sectional modeling. The relative and absolute contribution of variance component C was modeled to be a small contribution and is therefore not visible for most of the plot. Results of modeling in twins aged <20 y and ≥20 y are presented on a back-to-back basis, and the scale bar on the *y*-axis has been placed at *x* = 20 to mark the transition between the models. DMFS, decayed, missing, and filled surfaces.

In age-moderated analysis, no single quadratic term for age provided a good fit across the whole study group; however, when models were fitted separately in children and teenagers (*n* = 21,724; best-fitting term, age^2.15^) and adults aged ≥20 y (*n* = 17,756; best-fitting term, age^2.25^), stable models were identified with good concordance in estimates of heritability with the primary analyses and in fitted values at the crossover point between models (Fig. 2). The relative importance of shared environmental factors was modeled to decrease rapidly between ages 7 and 15 y and explain little variation in DMFS scores in older teenagers and adults. The estimated heritability peaked at nearly 60% in early adulthood before declining slightly with age but remained >50% across the adult life course.

### Clustering Analysis

**Figure 3.**
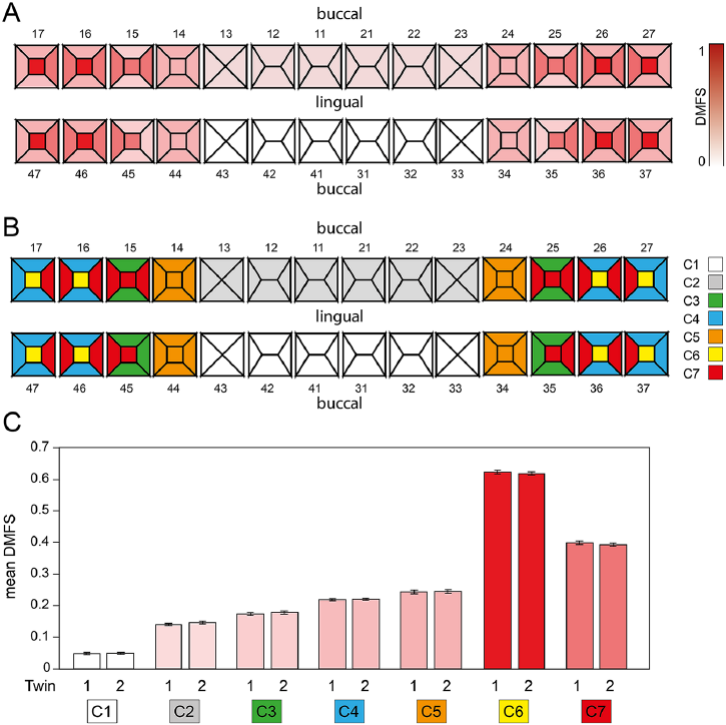
Surface-adjusted DMFS for caries clusters. (**A**) Mean per-surface DMFS in the 7 clusters identified via hierarchical clustering. (**B**) The 7 clusters (indicated in different colors) identified via hierarchical clustering. (**C**) Mean (95% CI) per-surface DMFS for each twin in the pair, where twin 1 was randomly selected for the cluster derivation data set and twin 2 was used for validation. The teeth are numbered by the ISO system per the World Health Organization notation system, adopted from the notation of the FDI, also called ISO 3950. It is a 2-digit numbering system in which the first digit represents a quadrant and the second digit represents the number of the tooth from the midline of the face. For permanent teeth, the upper right teeth begin with the number 1; the upper left teeth, the number 2; the lower left teeth, the number 3; and the lower right teeth, the number 4. DMFS, decayed, missing, and filled surfaces.

Unsupervised cluster analysis in adults identified a hierarchical pattern where tooth surfaces with similar anatomy formed nested clusters within broader functional units (Appendix Fig. 1). Seven clusters were identified that contained the same tooth surfaces in the derivation and validation data set, representing groups of tooth surfaces that are similar in their pattern of caries presentation (Fig. 3A–C). Cluster 1 includes lower incisors and canines; cluster 2, upper incisors and canines; cluster 5, all surfaces from the first premolars; cluster 3, all buccal and lingual surfaces of the second premolars and the mesial surfaces of the lower second premolar; cluster 4, all buccal and lingual surfaces of the molar and also the distal surfaces of the second molar; cluster 6, molar occlusal surfaces; and cluster 7, a mix of premolar and molar distal and mesial surfaces as well as the occlusal surfaces of the second premolar.

**Figure 4.**
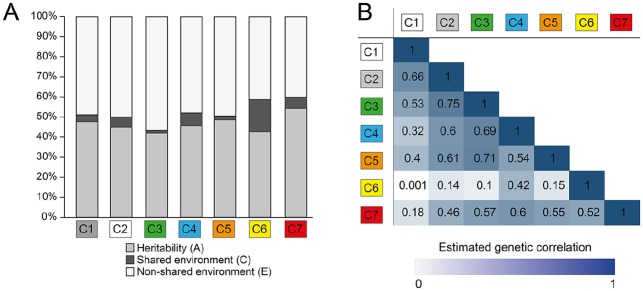
Results of variance decomposition for caries clusters. (**A**) Results of variance decomposition for each cluster and (**B**) the estimated genetic correlation (indicated by a color and a correlation coefficient) between each pair of caries clusters (indicated on the *x*- and *y*-axes).

With a multivariate ACE model to simultaneously model genetic and environmental contributions to each cluster, all clusters appeared moderately heritable, with the highest estimate for cluster 7 (54.3%) and the lowest estimate for cluster 3 (41.9%; Fig. 4A, Appendix Table 5). In general, shared environmental factors explained little variation in these clusters except for cluster 6, where 16.0% of variation was due to shared environmental influences.

Tooth surfaces may cluster because of differences in local (within-mouth) risk factors, or the relevance of environmental factors differ among tooth surfaces. Another potential explanation is that different genetic risk factors influence caries risk for different tooth surfaces, or the same genetic risk factors have surface-specific effects. To test this hypothesis, the genetic correlation among different clusters was estimated with the multivariate ACE model. No 2 clusters shared identical genetic factors (*P* values for difference in genetic determinants were significant for all comparisons); however, the genetic determinants of several clusters were moderately correlated—for example, clusters 2 and 3 with an estimated genetic correlation of 0.75 (95% CI, 0.69 to 0.81). The most distinct cluster was cluster 6, with a low genetic correlation with all other clusters (Fig. 4B, Appendix Table 5).

## Discussion

This study used a twin-based design nested in a national twin register to investigate the relative importance of genetic and environmental factors in caries. First, the study investigated summary indices for caries experience, finding that, similar to previous reports (Wang et al. 2010), genetic factors explain approximately 50% of variation in these scores but with more precise estimates made possible by the larger sample size of the present study.

Next the study tested the effects of genetic factors on caries progression, a topic that, apart from a 1-y follow-up in young children (Bretz, Corby, Schork, et al. 2005), is not covered by existing literature. Genetic effects explained at least 50% of variation in caries trajectories, with similar results from 2 modeling approaches and comparable results with a range of caries indices. Given that the study group represents a population with regular dental care, including preventative counseling and treatments, these findings suggest that genetic factors may also govern response to intervention strategies. Genome-wide association studies investigating caries progression in populations with access to dental care may provide insight into the biological processes underlying response or nonresponse to treatments and provide new ways to asses caries risk or improve treatment outcomes.

Finally, the study investigated the heritability of caries subtypes. Most studies consider the mouth as a unit with equal weighting in combined caries scores, though susceptibility and tooth-adjacent conditions are niche specific. Shaffer et al. (2013) reported 5 cluster-specific caries outcomes with similar but nonidentical membership in 2 family-based populations in the United States. The present study identified 7 clusters that were perfectly shared between the derivation and replication data sets, although this concordance may be partially due to the same families being present in both data sets. The difference in cluster patterns between the Swedish and US populations suggests that clustering might be affected by age, caries activity, or other factors within a population but is also subject to analytic decisions made by planning and interpreting cluster analysis.

The 7 clusters identified in the present study were all moderately heritable, with estimates ranging between 42% and 54%. For comparison, Shaffer et al. (2013) reported heritability estimates ranging between 0% and 54% for the different clusters, with the most heritable cluster representing anterior mandibular surfaces, which also had the lowest caries prevalence; however, these estimates had wide confidence intervals. In the present study, the genetic analysis indicated important differences in the etiology of the 7 clusters; for example, the cluster representing caries in occlusal surfaces of molars was more affected by shared environmental factors, possibly reflecting the earlier age of onset of caries at these surfaces as compared with other tooth surfaces. The genetic factors associated with the different clusters were only partially correlated. Importantly, differences in caries prevalence among clusters do not explain the weak genetic correlations between some clusters, as the heritability estimates and, therefore, genetic correlation estimates are standardized to the total phenotypic variation in each cluster. The incompletely overlapping genetic factors influencing the different clusters instead suggest that the clusters are affected by different genetic and biological factors or that the same genes have nonuniform effects on different clusters, which supports the idea that the clusters may be capturing biologically informative subtypes of disease. If present, these biological subtypes may be part of the explanation for why different tooth surfaces cluster into groups despite sharing the same macroenvironment of the mouth, although there may be other reasons for tooth surfaces to covary in clusters, such as cluster-specific environmental risk factors.

Investigating genetic associations with specific clusters may help uncover biological processes that are relevant for certain patterns of disease and may provide a bridge between understanding the causes of caries at a population level and understanding the biological processes leading to a patient presenting with a specific pattern of disease.

There are natural limitations to the twin-based approach and use of registry data—for example, dental data were obtained by many practitioners, and despite similar training, protocols, and standards, no formal calibration exercise was possible. If present, nonrandom measurement error, such as differences in diagnostic interpretation among dentists, might be fitted as a shared environmental factor (if twins go to the same dental office), while random measurement error would be fitted as a nonshared environmental factor in ACE modeling (Wray and Gottesman 2012), leading to an underestimation of heritability. Given that the estimates of heritability are comparable to or higher than those reported in studies with few examiners, it does not appear likely that measurement error had a major effect on these results. In common with other studies using the twin-based design, nonadditive or epistatic genetic effects, gene-environment correlation, or assortative mating may lead to a slight bias in heritability estimates. Unlike designs including twins reared apart, the present study relies on modeling to distinguish between shared environmental factors and additive genetic effects, and this limits investigation of dominant genetic effects (Verweij et al. 2012). The age-moderated analysis assumes that the only difference between younger and older participants in the study is due to age, but effects due to cohort or period may also be present. Finally, although the study represents twins at a range of ages (7 to 97 y), the findings relate only to permanent teeth and may not extrapolate to primary teeth.

In conclusion, host susceptibility to caries is likely to be governed by a collection of related genetic and molecular mechanisms that preferentially affect clusters of tooth surfaces rather than a few genetic mechanisms that are equally relevant to all teeth. Additional research is needed to identify the biologically causal genetic risk loci and explore ways to improve outcomes for people with high genetic risk for caries.

## Author Contributions

S. Haworth, contributed to conception, design, data analysis, and interpretation, drafted and critically revised the manuscript; A. Esberg, P. Lif Holgerson, contributed to data interpretation, drafted and critically revised the manuscript; R. Kuja-Halkola, contributed to data acquisition, analysis, and interpretation, critically revised the manuscript; N.J. Timpson, contributed to data interpretation, critically revised the manuscript; P.K.E. Magnusson, contributed to data acquisition and interpretation, critically revised the manuscript; P.W. Franks, contributed to conception, data acquisition, and interpretation, critically revised the manuscript; I. Johansson, contributed to conception, design, data acquisition, analysis, and interpretation, drafted and critically revised the manuscript. All authors gave final approval and agree to be accountable for all aspects of the work.

## Supplemental Material

DS_10.1177_0022034519897910 – Supplemental material for Heritability of Caries Scores, Trajectories, and Disease Subtypes Click here for additional data file. Supplemental material, DS_10.1177_0022034519897910 for Heritability of Caries Scores, Trajectories, and Disease Subtypes by S. Haworth, A. Esberg, P. Lif Holgerson, R. Kuja-Halkola, N.J. Timpson, P.K.E. Magnusson, P.W. Franks and I. Johansson in Journal of Dental Research
