# Supplementary material for: Heritability of Caries Scores, Trajectories, and Disease Subtypes
Source: J Dent Res. 2020 Jan 6;99(3):264–70. doi: 10.1177/0022034519897910 (PMC7036480; doi:10.1177/0022034519897910)

Heritability of Caries Scores, Trajectories, and Disease Subtypes

S. Haworth, A. Esberg, P. Lif Holgerson, R. Kuja-Halkola, N.J. Timpson, P.K.E. Magnusson, P.W. Franks, and I. Johansson

Appendix

Methods

Ethical approval

The STR received ethical approval from Regional Ethical Review Board in Stockholm, Sweden, Sweden. Analysis of caries traits received ethical approval from the local ethics committee at Umeå university, Sweden (Dnr 2010-387-31M with an approved addendum on heritability analyses May 23 2011). The study complied with all relevant ethical regulations including the Declaration of Helsinki.

Statistical modelling

In cross-sectional analysis the primary analysis used a single measure of DMFS, $DMFS_{proximal\ surfaces}$, DFS and $DFS_{proximal\ surfaces}$ for each individual. For convenience, the scores at the most recent dental examination were used. ACE models incorporated adjustment for age, age squared, birth year and sex as fixed effects. To aid model fit, all variables apart from sex were first standardized to have a mean of 0 and a standard deviation of 1. Model optimization used default parameters within OpenMX and results were only reported for models which reported a successful fit during optimization.

In age-stratified analysis twins were included if one twin in a pair fell up to 1 year outside the intended window. For example, if the twins in a pair were aged 19 and 20 at time of examination then that pair would be included in analysis of children and teenagers, but if the twins were aged 20 and 20 or 19 and 21 then the pair would be excluded.

To estimate whether the relative importance of genetic effects changes across the life course, additional models were fitted which omitted adjustment for age, age squared and birth year and instead fitted age as a moderator variable (Purcell 2002) where the magnitude of the A, C and E effects was allowed to vary with time. To optimize the fit of these models, the age variable was transformed using the equation $age_{trans}= age^{n}$ for a range of *n* values between 0.5 and 4.0, and the best fitting value of *n* was chosen by comparing the Akaike information criterion and Bayesian information criterion for each model. As the best fitting value of *n* was different in children and teenagers compared to adults, the decision was made to not fit these models in the entire dataset.

Parameters representing slope in caries scores with time (for the linear mixed model) and trajectory velocity, SITAR model) were extracted and modelled as an outcome in ACE models using the same approach as described for the univariate cross-sectional modelling.

Cluster analysis used a multivariate ACE model to simultaneously estimate the contribution of variance components A, C and E to variation in seven clusters. This approach allows the additive genetic effects contributing to each cluster to be compared using the genetic correlation statistic to estimate whether the different clusters have similar or distinct genetic determinants. Some of the cluster scores had markedly non-normal distribution. To help model identification, cluster scores were first regressed on age, age squared, birth year and sex, and the residuals

were transformed using an inverse normal rank transformation series. These transformed scores were then fitted in the ACE model with age, age squared, birth year and sex as fixed effects.

Estimation of variance attributable to lead single variants identified in genome-wide analysis.

First, the list of independent lead single variants for dental caries were obtained from the largest available genome-wide association study (Shungin et al. 2019). Effect allele frequency data and estimates of genetic effect on DMFS were obtained from Supplementary Dataset 3. These effect sizes are expressed in standard deviations of residuals after regression of DMFS on age, age squared, sex and other study specific covariates, meaning that the residuals can be treated the same as other standardized quantitative traits. Phenotypic variation due to each single nucleotide polymorphism (SNP) was estimated using the equation

$$\text{variation explained} \approx 2b\ 2f(1-f),$$

where b denotes the positively-signed effect estimate and f denotes the effect allele frequency, as previously described (Park et al. 2010).

These estimates ranged between 0.012% for rs149467613 to 0.082% for rs1122171. After estimating the variance attributable to each SNP, these were summed to estimate phenotypic variation due to all lead single variants, which was estimated at 1.4%.

References

Park J-H, Wacholder S, Gail MH, Peters U, Jacobs KB, Chanock SJ, Chatterjee N. 2010. Estimation of effect size distribution from genome-wide association studies and implications for future discoveries. Nature genetics. 42(7):570-575.

Purcell S. 2002. Variance components models for gene–environment interaction in twin analysis. Twin Research. 5(6):554-571.

Shungin D, Haworth S, Divaris K, Agler CS, Kamatani Y, Keun Lee M, Grinde K, Hindy G, Alaraudanjoki V, Pesonen P et al. 2019. Genome-wide analysis of dental caries and periodontitis combining clinical and self-reported data. Nature Communications. 10(1):2773.

Results

Appendix Table 1A. Participant characteristics by twin and zygosity in adult participants.

	Adults (20 years of age or older)					
	Twin 1			Twin 2		
Zygosity	MZ	DZSS	DZOS	MZ	DZSS	DZOS
Number[1]	n=3,309	n=2,736	n=3,385	n=3,300	n=2,758	n=3,367
Female[1], %	59	55.2	45.7	58.7	55.2	54.9
Age at dental visit	38.1 (37.5, 38.7)	42.1 (41.5, 42.8)	39.1 (38.5, 39.7)	38.1 (37.5, 38.6)	41.9 (41.2, 42.5)	39.2 (38.6, 39.8)
DMFS[2]	34.9 (34.1, 35.6)	36.2 (35.4, 36.9)	37.5 (36.8, 38.2)	34.7 (34, 35.4)	35.8 (35.1, 36.6)	37.4 (36.7, 38.1)
DMFSa[2]	14.5 (14.2, 14.9)	15.1 (14.7, 15.5)	15.8 (15.5, 16.1)	14.4 (14, 14.7)	14.9 (14.6, 15.3)	15.8 (15.4, 16.1)
DFS[2]	17.7 (17.2, 18.2)	19.1 (18.5, 19.6)	19.5 (19, 20)	17.5 (17.1, 18)	19.1 (18.6, 19.6)	19.5 (19, 19.9)
DFSa[2]	7.6 (7.3, 7.8)	8.2 (7.9, 8.5)	8.5 (8.2, 8.7)	7.4 (7.1, 7.7)	8.1 (7.8, 8.4)	8.5 (8.2, 8.7)

MZ=Monozygotic, DZSS = Dizygotic same sex, DZOS = Dizygotic opposite sex. 1) Numbers varies between twin 1 and twin 2 due to if they had their dental visit when they were still classified as <20 or ≥20 years of age. 2) adjusted for sex and age.

Appendix Table 1B. Participant characteristics by twin and zygosity in children and teenagers.

	Children/teenagers (7-age < 19 years old)					
	Twin 1			Twin 2		
Zygosity	MZ	DZSS	DZOS	MZ	DZSS	DZOS
Number[1]	n=3,061	n=3,558	n=4,790	n=3,070	n=3,536	n=4,808
Female[1], %	52.2	48.7	49.5	52.6	48.7	51.1
Age at dental visit	13.9 (13.8, 14.0)	13.5 (13.3, 13.6)	13.3 (13.2, 13.4)	13.9 (13.8, 14.1)	13.4 (13.3, 13.5)	13.3 (13.2, 13.4)
DMFS[2]	2.1 (2, 2.3)	2 (1.9, 2.2)	2.3 (2.1, 2.4)	2 (1.9, 2.2)	2.1 (2, 2.2)	2.2 (2.1, 2.3)
DMFSa[2]	0.6 (0.6, 0.7)	0.6 (0.5, 0.6)	0.7 (0.6, 0.7)	0.6 (0.5, 0.6)	0.6 (0.5, 0.7)	0.6 (0.6, 0.7)
DFS[2]	1.4 (1.3, 1.5)	1.4 (1.3, 1.5)	1.5 (1.5, 1.6)	1.4 (1.3, 1.5)	1.4 (1.3, 1.5)	1.5 (1.5, 1.6)
DFSa[2]	0.4 (0.3, 0.4)	0.3 (0.3, 0.4)	0.3 (0.3, 0.3)	0.3 (0.3, 0.3)	0.3 (0.3, 0.4)	0.4 (0.3, 0.4)

MZ=Monozygotic, DZSS = Dizygotic same sex, DZOS = Dizygotic opposite sex. 1) Numbers varies between twin 1 and twin 2 due to if they had their dental visit when they were still classified as <20 or ≥20 years of age. 2) adjusted for sex and age.

Appendix Table 2. Correlation in adjusted[1] caries indices in MZ and DZ twin pairs

Trait	Correlation coefficient (All ages)		Correlation coefficient (Age ≥20)		Correlation coefficient (Age < 20)	
	MZ	DZ	MZ	DZ	MZ	DZ
DMFS	0.54	0.28	0.54	0.27	0.58	0.28
DMFSa	0.58	0.32	0.58	0.31	0.55	0.24
DFS	0.59	0.36	0.58	0.36	0.68	0.39
DFSa	0.61	0.39	0.60	0.39	0.74	0.45
C1	-	-	0.40	0.42	-	-
C2	-	-	0.53	0.34	-	-
C3	-	-	0.49	0.28	-	-
C4	-	-	0.50	0.28	-	-
C5	-	-	0.52	0.25	-	-
C6	-	-	0.61	0.38	-	-
C7	-	-	0.59	0.32	-	-

MZ=Monozygotic, DZ=Dizygotic 1) For DMFS, DMFSa, DFS, and DFSa, scores were first adjusted for age, age squared, sex and birth year, and the residuals were used to estimate correlation coefficients. For caries clusters (C1-C7), the residuals were transformed using an inverse normal rank transformation prior to estimating correlation coefficient.

Appendix Table 3. Heritability estimates for each caries trait.

Analysis	Age group	A (95% CI)	P	C (95% CI)	P	E (95% CI)	P
DMFS	All ages	50.7 (47.5, 53.9)	2.6×10^{-215}	2.9 (0.6, 5.3)	0.015	46.4 (44.9, 47.9)	$<1 \times 10^{-300}$
	Age ≥20	56.8 (54.9, 58.6)	$<1 \times 10^{-300}$	3.9 (0.8, 7.0)	0.013	39.3 (35.6, 43.0)	1.0×10^{-96}
	Age <20	52.2 (50.2, 54.3)	$<1 \times 10^{-300}$	4.0 (0.2, 7.8)	0.038	43.8 (38.9, 48.7)	7.6×10^{-68}
DMFSa	All ages	51.2 (47.8, 54.7)	1.2×10^{-187}	6.2 (3.6, 8.8)	4.4×10^{-6}	42.6 (41.1, 44.0)	$<1 \times 10^{-300}$
	Age ≥20	61.4 (59.6, 63.2)	$<1 \times 10^{-300}$	0.5 (0-2.0)	0.47	38.0 (35.8, 40.3)	1.4×10^{-235}
	Age <20	49.1 (46.9, 51.1)	$<1 \times 10^{-300}$	7.2 (3.6, 10.7)	6.8×10^{-5}	43.8 (38.9, 49.0)	1.1×10^{-68}
DFS	All ages	50.0 (46.3, 53.7)	1.4×10^{-152}	11.0 (8.1, 14.0)	2.4×10^{-13}	39.0 (37.6, 40.4)	$<1 \times 10^{-300}$
	Age ≥20	62.7 (60.8, 64.8)	$<1 \times 10^{-300}$	0.1 (0-0.8)	0.88	37.1 (35.3, 39.1)	$<1 \times 10^{-300}$
	Age <20	61.0 (56.2, 65.8)	4.5×10^{-138}	5.9 (1.9, 9.9)	0.0035	33.1 (31.4, 34.8)	$<1 \times 10^{-300}$
DFSa	All ages	50.1 (46.8, 53.5)	1.9×10^{-190}	13.1 (10.5, 15.8)	1.1×10^{-22}	36.7 (35.4, 38.1)	$<1 \times 10^{-300}$
	Age ≥20	62.4 (58.2, 66.6)	9.4×10^{-189}	3.6 (0.2, 6.9)	0.037	34.0 (32.3, 35.8)	$<1 \times 10^{-300}$
	Age <20	58.7 (57.0, 60.3)	$<1 \times 10^{-300}$	3.2 (0- 7.1)	0.10	38.1 (33.9, 42.4)	8.3×10^{-70}

P values test the null hypothesis that the varance component (A, C or E) explains 0% of variation in the caries trait.

Appendix Table 4. Heritability estimates for longitudinal trajectory parameters.

Trajectory trait	Modelling approach	A (95% CI)	P	C (95% CI)	P	E (95% CI)	P
DMFS	LMM	56.9 (52.2, 61.7)	$6.1x10^{-123}$	4.8 (0.1, 8.7)	0.017	38.3 (36.7, 39.9)	$<1x10^{-300}$
	SITAR	58.5 (53.6, 63.4)	$1.9x10^{-118}$	0.1 (0, 4.8)	0.76	40.8 (39.2, 42.5)	$<1x10^{-300}$
DMFSa	LMM	59.5 (54.8, 64.1)	$3.2x10^{-137}$	3.3 (0-7.2)	0.095	37.2 (35.6, 38.8)	$<1x10^{-300}$
	SITAR	55.8 (50.7, 60.9)	$8.2x10^{-104}$	1.4 (0-5.6)	0.49	42.7 (41.0, 44.5)	$<1x10^{-300}$
DFS	LMM	60.1 (56.1, 64.1)	$6.3x10^{-190}$	10.9 (7.5, 14.4)	$5.7x10^{-10}$	28.9 (27.6, 30.3)	$<1x10^{-300}$
	SITAR	52.2 (47.7, 56.7)	$6.6x10^{-116}$	11.9 (8.2, 15.7)	$2.9x10^{-10}$	35.9 (34.3, 37.4)	$<1x10^{-300}$
DFSa	LMM	60.5 (56.2, 64.6)	$1.3x10^{-175}$	8.3 (4.7, 11.8)	$5.0x10^{-6}$	31.3 (29.9, 32.7)	$<1x10^{-300}$
	SITAR	50.0 (45.5, 54.5)	$1.9x10^{-106}$	13.7 (10.0, 17.4)	$5.1x10^{-13}$	36.3 (34.7, 37.8)	$<1x10^{-300}$

P values test the null hypothesis that the varance component (A, C or E) explains 0% of variation in the trajectory paramater.

Appendix Table 5. Heritability estimates for each caries cluster.

Cluster	A (95% CI)	P	C (95% CI)	P	E (95% CI)	P
C1	47.5 (42.1, 52.9)	$7.6x10^{-67}$	3.5 (0-7.4)	0.08	49.0 (46.5, 51.4)	$<1x10^{-300}$
C2	44.8 (39.5, 50.0)	$8.1x10^{-63}$	4.9 (0.95, 8.9)	0.015	50.2 (47.9, 52.7)	$<1x10^{-300}$
C3	41.9 (38.5, 45.3)	$1.9x10^{-127}$	1.4 (0-3.6)	0.19	56.7 (54.3, 59.1)	$<1x10^{-300}$
C4	45.9 (40.7, 51.0)	$4.2x10^{-69}$	6.1 (2.2, 10.0)	0.0022	48.0 (45.7, 50.3)	$<1x10^{-300}$
C5	48.7 (45.5, 52.0)	$3.3x10^{-190}$	1.7 (0-3.9)	0.13	49.5 (47.4, 51.6)	$<1x10^{-300}$
C6	42.5 (37.0, 48.0)	$2.6x10^{-51}$	16.0 (11.5, 20.6)	$3.2x10^{-12}$	41.4 (69.4, 43.4)	$<1x10^{-300}$
C7	54.3 (49.4, 59.2)	$5.8x10^{-104}$	5.7 (1.6, 9.7)	0.0058	40.0 (38.1, 42.0)	$<1x10^{-300}$

P values test the null hypothesis that the varance component (A, C or E) explains 0% of variation in the caries cluster.

Appendix Table 6. Estimated genetic correlation between different caries clusters

	Estimated genetic correlation (SE) [P*]					
**Cluster**	**C1**	**C2**	**C3**	**C4**	**C5**	**C6**
**C2**	0.66 (0.029) [$1.7x10^{-33}$]	1				
**C3**	0.53 (0.041) [$2.8x10^{-30}$]	0.75 (0.031) [$3.6x10^{-16}$]	1			
**C4**	0.32 (0.042) [$5.9x10^{-59}$]	0.60 (0.028) [$3.6x10^{-47}$]	0.69 (0.027) [$1.7x10^{-31}$]	1		
**C5**	0.40 (0.030) [$2.0x10^{-87}$]	0.61 (0.023) [$2.6x10^{-61}$]	0.71 (0.020) [$6.4x10^{-47}$]	0.54 (0.025) [$8.0x10^{-74}$]	1	
**C6**	0.00056 (0.047) [$2.4x10^{-101}$]	0.14 (0.047) [$1.3x10^{-75}$]	0.10 (0.047) [$9.5x10^{-82}$]	0.42 (0.042) [$3.4x10^{-44}$]	0.15 (0.041) [$1.3x10^{-95}$]	1
**C7**	0.18 (0.039) [$2.8x10^{-97}$]	0.46 (0.032) [$1.4x10^{-65}$]	0.57 (0.023) [$2.3x10^{-77}$]	0.60 (0.021) [$2.1x10^{-83}$]	0.55 (0.035) [$3.1x10^{-94}$]	0.52 (0.035) [$3.1x10^{-43}$]

Table contents are genetic correlation estimates, with standard errors in brackets.
P values in square brackets test the null hypothesis that the genetic correlation=1.

Appendix Figure 1. Dendrogram in one randomly selected adult twin.
The teeth are numbered by the ISO system by the World Health Organization notation system, adopted from the notation of the FDI, also called ISO 3950. It is a two-digit numbering system in which the first digit represents a quadrant and the second digit represents the number of the tooth from the midline of the face. For permanent teeth, the upper right teeth begin with the number, "1". The upper left teeth begin with the number, "2". The lower left teeth begin with the number, "3" and the lower right teeth begin with the number, "4".

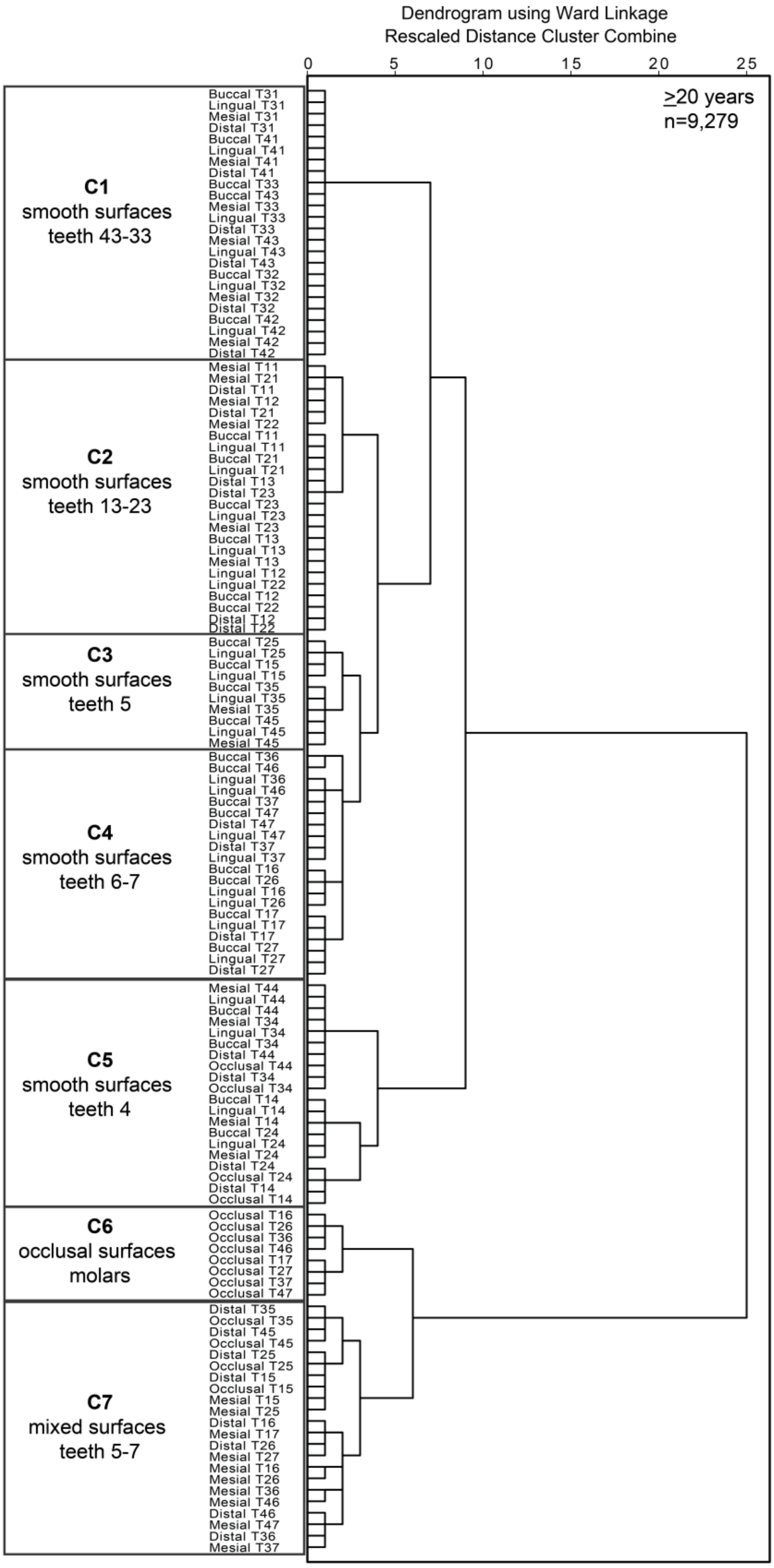

Supplement: DS_10.1177_0022034519897910 – Supplemental material for Heritability of Caries Scores, Trajectories, and Disease Subtypes [file DS_10.1177_0022034519897910.pdf]
